# Childhood obesity and dental caries: an ecological investigation of the shape and moderators of the association

**DOI:** 10.1186/s12903-020-01329-7

**Published:** 2020-11-25

**Authors:** Vahid Ravaghi, Amir Rezaee, Miranda Pallan, Alexander John Morris

**Affiliations:** 1 School of Dentistry, University of Birmingham, 5 Mill Pool, Birmingham, B5 7EG UK; 2 International Business School, 8 rue de Lota, 75116 Paris, France; 3 Institute of Applied Health Research, University of Birmingham, Birmingham, UK

**Keywords:** Obesity, Decay, Children, Teeth

## Abstract

**Background:**

Despite sharing a common risk factor in dietary sugars, the association between obesity and dental caries remains unclear. We investigated the association between obesity and dental caries in young children in England in an ecological study.

**Methods:**

We analysed data from 326 lower tier English local authorities. Data on obesity and dental caries were retrieved from 2014/15 to 2016/17 National Child Measurement Programme and 2016/17 National Dental Epidemiology Programme. We used fractional polynomial models to explore the shape of the association between obesity and dental caries. We also examined the modifying effect of deprivation, lone parenthood, ethnicity, and fluoridation.

**Results:**

Best fitting second order fractional polynomial models did not provide better fit than the linear models for the association between obesity and prevalence and severity of dental caries; therefore, the linear model was found suitable. Despite significant association, after adjusting for the effect of deprivation, obesity was neither associated with prevalence (coefficient = 0.2, 95% CI − 0.71, 0.75), nor with severity (coefficient = 0.001, 95% CI − 0.03, 0.03) of dental caries. In fully adjusted models, the proportion of white ethnicity and being in fluoridated areas were associated with a decrease in dental caries. The association between obesity and dental caries was moderated by the effect of deprivation, white ethnicity, and lone parenthood.

**Conclusions:**

The association between obesity and dental caries was linear and moderated by some demographic factors. Consequently, interventions that reduce obesity and dental caries may have a greater impact on specific groups of the population.

## Introduction

Childhood obesity is one of the most serious global public health challenges, with mean Body Mass Index (BMI) increasing globally in past decades in most regions [1]. Children with overweight and obesity are at greater risk of obesity in adulthood and developing related conditions such as diabetes and cardiovascular diseases at a younger age [2]. Despite a declining prevalence seen in westernised high income countries, dental caries also continues be an important global public health problem [3]. In England, around a quarter of children aged 4 to 5 years of age are living with overweight or obesity [4] and a similar proportion (23%) of children aged 5 years have experienced dental caries [5]. Like obesity, dental caries has a high impact on individuals and health services, for example the majority of admissions to hospital for children in England in the period 1997–2006 were primarily due to dental caries [6]. In England, both obesity and dental caries share a pro-poor association with social deprivation, with children from more deprived backgrounds being at greater risk [4, 5].

Dietary intake is a risk factor for both dental caries and obesity; as a result, it is believed that modifying dietary practices may be an effective intervention to reduce both dental caries and obesity [7]. This approach has been one of the pillars of dental health policy in England [8] and assumes that dental caries and obesity in children are associated and share common risk factors. While some systematic reviews found no association [9, 10], two systematic reviews found some evidence in favour of an association under specific conditions [11, 12]. For example, Hayden and colleagues reported a significant association between children obesity and dental caries only in industrialised countries [12]. Others reported an association at around the age of 5 to 9 years but not in younger children [11].

Reducing sugar consumption among children is now a major health policy in England, seeking to achieve 20% reduction in children consumption of sugar by 2020; the main driver of this policy is childhood obesity prevention [13]. With sugar being labelled as the ‘single cause’ of dental caries [14], these policies are likely also to influence dental health. A policy document titled “Sugar reduction: the evidence for action”, which outlines sugar reduction policies in England, has estimated that nearly a quarter a million cases of dental caries will be avoided following a 5% reduction in energy intake from sugar [15].

A better understanding of the nature of the association between childhood obesity and dental caries will help to predict the impact of current policies on the prevalence of dental caries and childhood obesity. Therefore, this study aims to investigate, from a new perspective, the association between dental caries and obesity among young children. We set out to examine the shape of the association between obesity and dental caries; whether it is linear or non-linear. Secondly, we aimed to explore whether this association varies by other characteristics of the population: deprivation, ethnicity, proportion of lone parents and living in a fluoridated area.

## Methods

Data for this study were retrieved from both survey and administrative data sources. Data on dental caries for English lower tier local authorities (LA) were retrieved from the 2016/17 oral health survey of 5-year-old children. The detailed findings of this survey has been previously reported [5]. Dental caries in each LA was indicated by its prevalence and severity. The proportion of children who had experienced dental caries in each LA indicated prevalence and the mean number of decayed, missing and filled teeth (dmft) indicated severity.

Average prevalence of obesity among children in reception class (age 4–5 years) in each local authority for the years 2014/15 to 2016/17 was retrieved from the National Child Measurement Programme (NCMP) [16]; in which the obesity was defined as children with a body mass index (BMI) greater than or equal to the 95th centile of the British 1990 growth reference BMI distribution have been classified as obese. Deprivation status of LAs was determined using by the average scores for deprivation as reported in the 2015 Index of Multiple Deprivation [17]. Percentages of lone parents with dependent children and white ethnicity (English/Welsh/Scottish/Northern Irish/British) in each LA were retrieved from the latest UK census which was carried out in 2011 [18]. The number of children attending NHS dentists in each LA were obtained from NHS Digital Statistics through a Freedom of Information request. Dental attendance of children aged 0–5 was calculated using the number of 0–5 year old children seen by the end of June 2017 and the 2016 mid-year population and was obtained through freedom of information request [19]. Local authorities where some of the population receive a water supply with adjusted fluoride levels were identified from the 2018 Public Health England (PHE) report ‘Water fluoridation; Health monitoring report for England’ [20].

To explore the shape of the association between dental caries and obesity, we tested the null hypothesis of linearity against alternative regression functions and selected the best fitting model. In doing so, we used fractional polynomial regression models. This technique, proposed by Royston and Sauerbrei [21], evaluates whether the effect of a continuous variable (i.e. obesity rate) on the outcome (i.e. prevalence of dental caries) is better modelled by a nonlinear fractional polynomial (FP) function. FPs are of the form:

$$Y = B0 + B{1} \cdot X{\text{p1}} + B{2} \cdot X{\text{p2}} + \cdots$$

where p1, p2, … are selected from default set of powers {− 2, − 1, − 0.5, 0, 0.5, 1, 2, and 3} with 0 signifying logarithm of variable. In this formula, Y represents the dental caries outcome (e.g. Mean dmft score), *B*0 is the intercept, and X represents prevalence of obesity. The *B*1 and *B*2 coefficients capture the effect of first and second orders for deprivation ranking, respectively. Conventionally, fractional polynomial models that involve two terms (i.e. first and second orders) are assumed to be adequate for identifying the best fit [22]. Therefore, we fitted 44 models for each dental caries outcome with the combinations of powers fitted; out of those we reported the statistical estimates for the linear model and first order (m = 1) and best fitting second order models (m = 2) for the abovementioned default set of powers:

$${\text{Dental}}\;{\text{caries}} = B0 + B{1}\;{\text{Obesity}}^{{{\text{p1}}}} + B{2}\;{\text{Obesity}}^{{{\text{p2}}}}$$

We selected the best fit model between selected models based on the algorithm suggested by Royston and Altman [22] which estimates the deviance where deviance is defined as twice the negative log likelihood. In addition to deviance, we reported the estimates of the residual standard errors and the *p* values for the partial F test comparing models’ deviance. As such, we initially compared the best fitting second order model (m2) with the linear model; if this did not provide a statistically better fit, the linear model was selected. Otherwise, the best fitting second order model (m2) was compared with the best fitting first-order model (m1). The best fitting second order model (m2) was preferred to first order model (m1) only if it provided a statistically better fit; otherwise, the first order model (m1) was selected. This approach allowed us to choose the simplest as well as best fitting model [23]. We used the STATA ‘fracpoly’ command to produce and compare fractional polynomial models.

To address the second objective of this study, we added interaction terms to final regression models in order to establish whether the association between obesity and dental caries varies according to other characteristics such as: deprivation, ethnicity, and lone parenthood, and living in fluoridated area. We estimated average marginal effects after adjusting for other covariates.

## Results

We analysed data for 303 out of 326 lower-tier LAs as these had data on dental caries. Descriptive statistics have been reported in Table 1. When we fitted the linear model for both prevalence and severity of dental caries, obesity explained 26% of the variation for prevalence of dental caries and mean dfmt. We then fitted additional models with quadratic and cubic terms to permit one and two bends in the association between both dental caries outcomes and obesity. The R-squared showed a marginal increase (less than 1%) for either of quadratic and cubic models; therefore, these models did not provide an evidence for departure from linearity (data not shown).

**Table 1:** Descriptive statistics

Variable	N	Mean^a^	Std. Dev	Range
Mean dmft score (decayed, missing, filled teeth)	302^b^	0.7	0.4	(0.1, 2.3)
Prevalence of dental caries (% d_3_mft > 0)	302	21.7	8.7	(4.4, 49.4)
Index of multiple deprivation (average score)	324	19.5	8.0	(5.0, 42.0)
Obesity (%)	324	8.9	1.7	(4.2, 13.7)
Lone parenthood in population (%)	324	6.6	1.7	(4.0, 14.4)
White population (%) (English/Welsh/Scottish/Northern Irish/British)	324	84.3	16.4	(16.7, 97.6)
Dental attendance rate	324	45.9	8.5	(19.5, 68.7)
Fluoride (0 = not fluoridated; 1 = fluoridated)	324	N/A	N/A	(0, 1)

^a^These estimates do not take into account population weight. Therefore, they do not represent national estimates in other official reports

^b^In total, 303 LAs participated in the survey but data from one LA (city of London) were merged with another LA (Hackney)

**Fig. 1:**
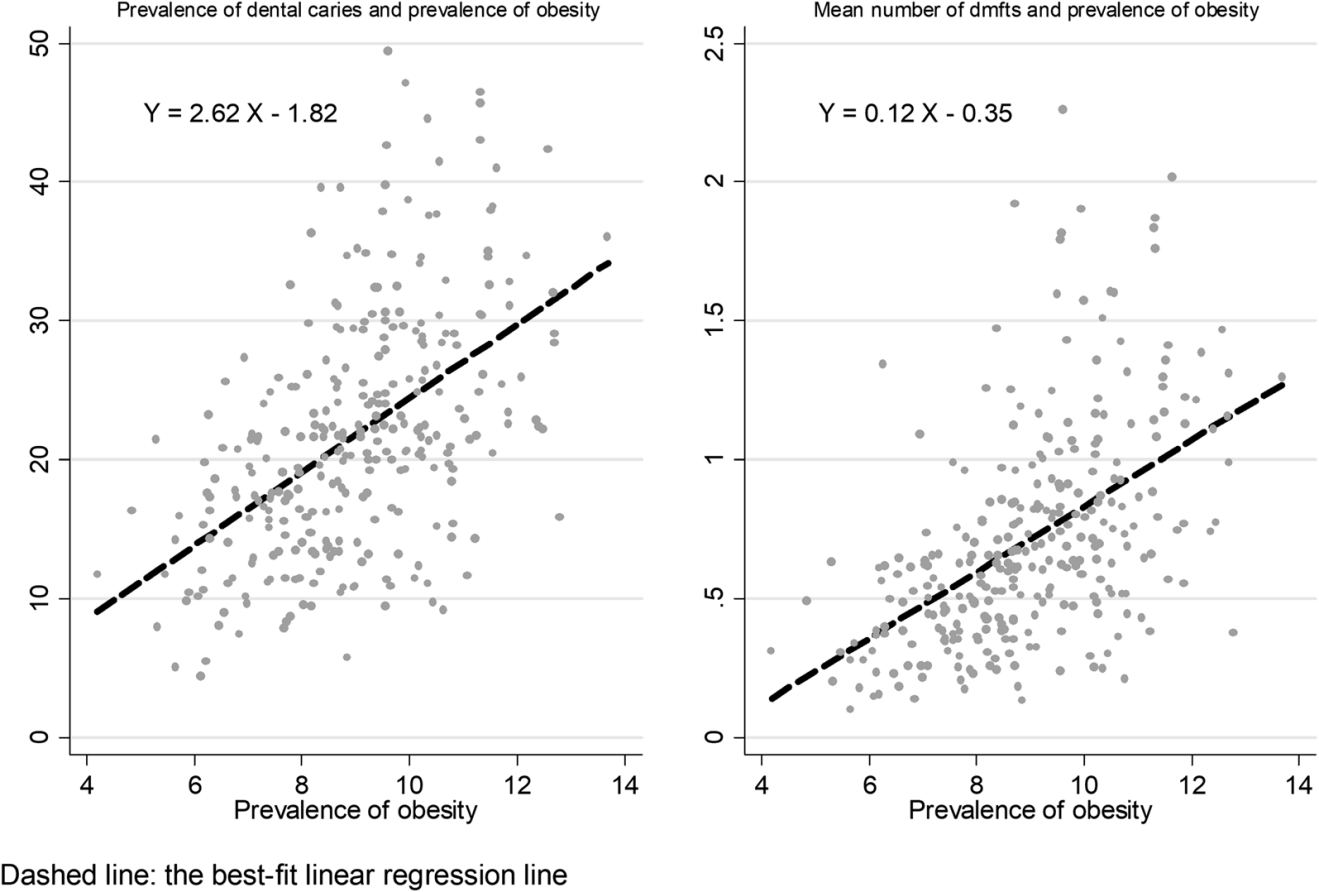
Dental caries and prevalence of obesity in English local authorities

Table 2 demonstrates the estimates from fitting first- and second order polynomials. Values of ‘deviance difference’ for linear and first order model (m = 1) represent the extent to which second order model (m = 2) is, comparatively, a better fit. Examining the shape of the association between prevalence of dental caries and obesity, the best-fitting first-order (m = 1) polynomial model had a power 0.5 for obesity, whereas the best-fitting second-order polynomial (m = 2) had powers (− 2 − 1). However, the second order model was not significantly better in terms of model fit to data than the linear model (deviance difference = 1.75, *p* = 0.627) or the first order model (m = 1) (deviance difference = 1.57, *p* = 0.46). For mean dmft scores, the best fitting first and second order polynomials had powers 1 and (− 2 − 1), respectively. Again, there was no statistically significant difference to justify using fractional polynomial models, as they did not have any better fit than a simple linear model. As fractional polynomial models had no better fit than a simple linear model, we therefore selected linear models for final analyses. The scatterplot and the best fitting linear regression line are displayed in Fig. 1.

**Table 2:** Comparison of linear and non-linear fractional polynomial regression models for dental caries

	Degree of freedom	Deviance	Res. SD	Deviance difference	*p* values	Powers
Prevalence of dental caries (% d_3_mft > 0)						
Linear	1	2066.51	7.43	1.75	0.627	1
m = 1	2	2066.33	7.43	1.57	0.46	0.5
m = 2	4	2064.76	7.42	–	–	− 2, − 1
Mean dmft score (Decayed, missing, filled teeth)						
Linear	1	192.23	0.33	1.57	0.669	1
m = 1	2	192.23	0.33	1.57	0.461	1
m = 2	4	190.66	0.33	–	–	− 2, − 1

Table 3 shows the results of the linear regression models adjusted for covariates. While obesity was directly related to indicators of dental caries in the unadjusted linear regression model (model 1), it was neither associated with prevalence (coefficient = 0.2, 95% CI − 0.71, 0.75), nor with severity (coefficient = 0.001, 95% CI − 0.03, 0.03) of dental caries in model 2 which was adjusted for the effect of deprivation. When we adjusted the model for the effect of all covariates (model 3), the coefficient of obesity remained non-significant; while greater deprivation and dental attendance were associated with higher prevalence of dental caries and mean dmft. In addition, the dental caries outcomes were inversely related to proportion of white ethnicity and being in fluoridated area.

**Table 3:** Linear regression coefficients and their confidence intervals for two indicators of dental caries

	Prevalence of dental caries (% d_3_mft > 0)			Mean dmft score (Decayed, missing, filled teeth)		
	Model 1 Coefficient (95% CI)	Model 2 Coefficient (95% CI)	Model 3 Coefficient (95% CI)	Model 1 Coefficient (95% CI)	Model 2 Coefficient (95% CI)	Model 3 Coefficient (95% CI)
Obesity	2.62*** (2.12, 3.12)	0.02 (− 0.71, 0.75)	0.3 (− 0.38, 0.97)	0.12*** (0.10, 0.14)	0.001 (− 0.03, 0.03)	0.02 (− 0.01, 0.05)
Deprivation		0.69*** (0.54, 0.85)	0.70*** (0.53, 0.87)		0.03*** (0.02, 0.04)	0.03*** (0.03, 0.04)
Dental attendance			0.39*** (0.29, 0.49)			0.02*** (0.01, 0.02)
Lone Parenthood			− 0.51 (− 1.24, 0.22)			− 0.04** (− 0.08, − 0.01)
White ethnicity			− 0.20*** (− 0.26, − 0.14)			− 0.01*** (− 0.01, − 0.01)
Fluoridation (0 = not fluoridated; 1 = fluoridated)			− 2.30* (− 4.56, − 0.04)			− 0.18*** (− 0.28, − 0.08)
(Constant)	− 1.82 (− 6.36,2.71)	8.01*** (3.42,12.60)	7.66* (0.63, 14.70)	− 0.35*** (− 0.55, − 0.14)	0.1 (− 0.10, 0.31)	0.3 (− 0.00, 0.61)
N	302	302	302	302	302	302

95% confidence intervals in brackets (*p < 0.05; **p < 0.01; ***p < 0.001)

Model 1 was unadjusted

Model 2 was adjusted for deprivation

Model 3 was adjusted for all covariates (deprivation, dental attendance, lone parenthood, white ethnicity, fluoridation)

To assess the effect of modifying variables, we added the interaction terms to final models. For the prevalence of dental caries, there was significant interaction between obesity and three covariates: deprivation (*p* = 0.023), white ethnicity (*p* < 0.001), and lone parenthood (*p* = 0.001). For mean dmft score, interaction effect was significant for two covariates: white ethnicity (*p* < 0.001) and lone parenthood (*p* = 0.008). The interaction effect between obesity and fluoridated area was not significant for either indicator of dental caries. Table 4 shows the marginal effects of dental caries outcomes for different values of moderators (i.e. 1st Percentile, 25th Percentile, 50th Percentile and 75th Percentile). Marginal effects estimated the amount of change in the predicted values of outcome variable for one unit change in obesity adjusted for other covariates. Table 4 shows that the change in dental caries increases with an increase in obesity rate across different levels of modifiers. The association between obesity and dental caries was steeper in less deprived areas and those areas with a smaller proportion of lone parents. There was an inverse association between dental caries and obesity in local authorities with a lower proportion of white ethnicity, unlike areas with a higher proportion of white population.

**Table 4:** Marginal effects for different values of modifiers

	Prevalence of dental caries (% d_3_mft > 0)		Mean dmft score (Decayed, missing, filled teeth)	
	Marginal effects (95% CI)	*p* values	Marginal effects (95% CI)	*p* values
Deprivation^a^				
1st percentile	0.83* (0.02, 1.65)	0.046	N/A	N/A
25th percentile	0.52 (− 0.18, 1.21)	0.147	N/A	N/A
50th percentile (Median)	0.23 (− 0.44, 0.91)	0.502	N/A	N/A
75th percentile	− 0.16 (− 0.94, 0.62)	0.686	N/A	N/A
White ethnicity				
1st percentile	− 2.79 *** (− 4.2, − 1.37)	*p* < 0.001	− 0.11 ** (− 0.17, − 0.05)	0.001
25th percentile	0.3* (− 0.36, 0.95)	0.370	0.02 (− 0.01, 0.05)	0.224
50th percentile (Median)	0.87* (0.17, 1.56)	0.014	0.04 ** (0.01, 0.07)	0.008
75th percentile	1.14** (0.4, 1.88)	0.003	0.05 ** (0.02, 0.09)	0.001
Lone parenthood				
1st percentile	0.81* (− 0.05, 1.67)	0.066	0.03 (− 0.01, 0.07)	0.120
25th percentile	0.45 (− 0.3, 1.2)	0.239	0.02 (− 0.01, 0.05)	0.261
50th percentile (Median)	0.06 (− 0.65, 0.78)	0.868	0.01 (− 0.03, 0.04)	0.670
75th percentile	− 0.36 (− 1.15, 0.43)	0.368	− 0.01 (− 0.04, 0.03)	0.722

*N/A* not applicable due to non-significant interaction terms

95% confidence intervals in brackets (*p < 0.05; **p < 0.01; ***p < 0.001)

^a^There was no interaction between deprivation and mean dmft scores, therefore corresponding marginal effects have not been reported

## Discussion

We found a linear association between obesity and dental caries in bivariate analyses; however, this association disappeared after accounting for the effect of deprivation. Furthermore, we showed that the association between obesity and dental caries was not uniform; rather, the strength and direction of this association varies by modifying characteristics such as deprivation, ethnicity and lone parenthood. The findings of our study are relevant to oral health policies aiming to reduce dental caries and obesity through the common risk factor approach.

We did not establish an independent association between prevalence of childhood obesity and dental caries in English local authorities. Previously, an official PHE report suggested a ‘weak to moderate’ association between dental caries and obesity prevalence in children [24]. This report, however, did not examine the independent association between obesity and dental caries after taking into account the effect of confounding variables such as deprivation. Nevertheless, the lack of an association between childhood obesity and dental caries in this study could be explained in a number of ways. Most notably, consumption of free sugar is a risk factor both dental caries and obesity; however, obesity could also occur as a result of excessive consumption of non-cariogenic foods. This may raise questions about use of the common risk factor approach in tackling dental decay, a point which has been highlighted by other authors [25]. Our findings regarding the influence of deprivation, the prevalence of lone parenthood and white ethnicity on the association between obesity and dental caries prevalence suggests that a more nuanced understanding of dietary intake patterns in different population groups is required which, in turn, would inform prevention approaches to both obesity and caries.

Previous studies have rarely assumed the possibility of non-linear association between childhood obesity and dental caries. Using fractional polynomial modelling, we did not find a non-linearity in the association between childhood obesity and dental caries. It should be noted, however, that the findings of our study were obtained from analyses of area-level rather than individual-level data. To our knowledge, one study at least has shown a U-shaped association between dental caries and weight with those being either underweight or obese reporting higher dental caries than normal weight children [26]. It has been proposed that some of the inconsistencies in the literature regarding the association between children dental caries and obesity could be due to dismissing the non-linear shape of the association between two health conditions [11]. Again, having a good understanding of this association will enable a more tailored approach to understanding risk factors and developing obesity and caries prevention approaches for children of different weight status.

While obesity was not significantly associated with dental caries in our fully adjusted models, we showed that deprivation, lone parenthood and ethnicity moderated the association. Of particular note is that obesity was more strongly related to the prevalence of dental caries in the least deprived areas while there was a weak or no association in more deprived areas. The interrelationship between obesity, dental caries and socioeconomic status has been a common observation although the reasons behind this are complex and poorly understood [9]. In concordance with our findings, others have also showed a more pronounced association between obesity and dental caries in more affluent children [27] and countries [11, 12]. This notion has important policy implications; a population approach that is successful in reducing both obesity and dental caries prevalence may have a greater impact on caries in the less deprived communities compared with the more deprived communities and may inadvertently widen oral health inequalities. The risk that some public health interventions may increase inequalities in health outcomes is well-documented [28] and should be a significant consideration in designing health policy.

We also showed that ethnicity moderates the association between obesity and dental caries. Unlike areas with a predominantly white population, in areas with a majority non-white population there was an inverse association between obesity and dental caries. There is evidence to show that children of white ethnicity have a higher dietary intake of sugar compared to other ethnic groups in the UK, which may partly explain this finding [29].

Our study, for the first time, used national data from England to examine the link between obesity and dental caries at local authority level. While there have been many cross-sectional studies to examine the link between obesity and dental caries, ecological studies, which enable exploration of associations on a larger scale, have been scarce. The findings of our study, however, should be interpreted in the light of limitations of an ecological study design and may not be extrapolated at the sub-population or individual level.

## Conclusion

The relationship between childhood obesity and dental caries is complex and moderated by socio-demographic factors. Health policies aiming to reduce obesity and dental caries through the common risk factor approach need to account for the complexity of this relationship and the effect of socio-demographic modifiers.

## Abbreviations

BMI: Body mass index
FP: Fractional polynomial
LA: Local authority
NCMP: National Child Measurement Programme
PHE: Public Health England
